# Trifluoromethylated proline analogues as efficient tools to enhance the hydrophobicity and to promote passive diffusion transport of the l-prolyl-l-leucyl glycinamide (PLG) tripeptide†

**DOI:** 10.1039/c8ra02511h

**Published:** 2018-04-18

**Authors:** Martin Oliver, Charlène Gadais, Júlia García-Pindado, Meritxell Teixidó, Nathalie Lensen, Grégory Chaume, Thierry Brigaud

**Affiliations:** Laboratoire de Chimie Biologique (LCB), Université de Cergy-Pontoise 5 mail Gay-Lussac, Neuville-sur-Oise 95031 Cergy-Pontoise France nathalie.lensen@u-cergy.fr gregory.chaume@u-cergy.fr; Institute for Research in Biomedicine (IRB Barcelona), Barcelona Institute of Science and Technology (BIST) C/ Baldiri Reixac 10 08028 Barcelona Spain

## Abstract

The synthesis of four CF_3_-proline analogues of the PLG peptide is reported. Our results show that the incorporation of trifluoromethylated amino acids (Tfm-AAs) at the N-terminal position of a peptide significantly increases its hydrophobicity. In addition, depending on the relative configuration and the position of the CF_3_ group, Tfm-AAs can also promote passive diffusion transport.

## Abstract 2

The incorporation of trifluoromethylated proline analogues in the tripeptide PLG enhances its hydrophobicity and promotes passive diffusion transport.

## Graphical abstract

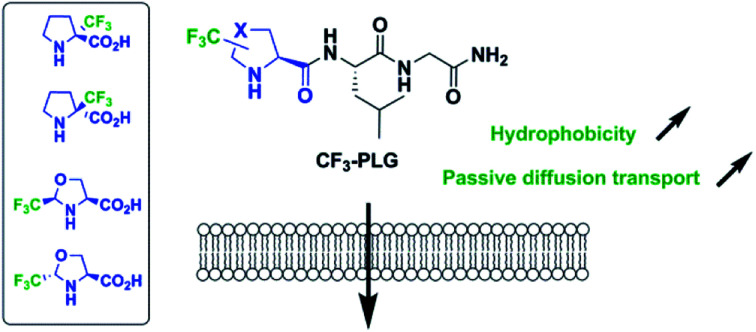

## Introduction

The use of peptides as therapeutic agents are gaining interest and the number of peptides entering into clinical trials is in steady increase.^1^ Nonetheless their development is hampered by the lack of metabolic stability, poor transport properties, low oral bioavailability as well as poor target specificity. Therefore, efforts are still needed to circumvent these drawbacks and the incorporation of constrained unnatural amino acids into the peptide chains is one of the common strategies to improve their biological profile. In this context, fluorinated amino acids are very attractive compounds.^2^ The introduction of fluorine atoms and fluoroalkyl groups into peptides and peptidomimetics presents the following advantages: (i) it can greatly increase the local hydrophobicity,^3^ it also modulates the peptide conformation and assembly,^4^ (ii) it confers a better resistance to proteolysis^5^ and (iii) fluorine atoms can be used as highly sensitive labels for ^19^F-NMR/MRI/MRS spectroscopy, because of ^19^F 100% natural abundance and the absence of background signals in natural and biological media.^6^ Unfortunately, the use of fluorinated peptides remains limited, especially for amino acids bearing a trifluoromethyl group adjacent to the amino group such as trifluoromethylated amino acids (Tfm-AAs). Indeed, only few synthetic methods are available for their synthesis in enantiopure form resulting in a small panel of fluorinated amino acids. Moreover, their incorporation into peptides is still challenging due to the stereoelectronic effects imparted by the CF_3_ group which strongly decrease the nitrogen atom nucleophilicity^7^ and limits the coupling reactions at their N-termini.

Our group develops for several years efficient methods for the synthesis of Tfm-AAs^8^ and their incorporation into peptides.^9^ We have reported that, unlike the non-fluorinated series, the coupling reactions at the C-termini of Tfm-AAs can be achieved with unprotected amino group because of its strong deactivation by the electron-withdrawing effect of the neighbouring CF_3_ group.^9*a*^ In contrast, the decrease of the nucleophilicity of the amino group together with the steric hindrance of the CF_3_ group significantly impedes the coupling reaction of Tfm-AAs at their N-terminus and specific activations such as mixed anhydrides or amino acid chlorides have to be used.^9*b*,*c*,10^ We applied our methodologies to the synthesis of several Tfm-AAs containing peptides in order to investigate their specific physicochemical and/or biological properties.^11^

**Fig. 1:**
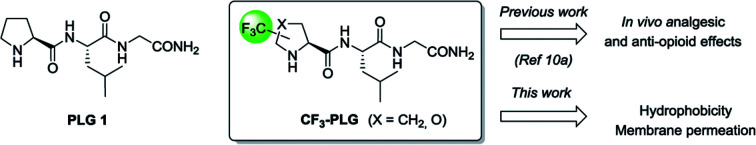
Chemical structure of PLG and CF_3_-PLG analogues.

Among the targeted peptides, we are interested in the endogenous brain peptide l-prolyl-l-leucyl glycinamide 1 (PLG). Because of its structural simplicity, and its relevance in a wide range of pharmacological activities in the central nervous system (CNS),^12^ several conformationally constrained analogues of PLG have been reported in literature.^13^ From our side, we have previously described the synthesis of the (*S*)-α-CF_3_-proline analogue (*S*,*S*)-2 of PLG. *In vivo* biological activity studies on rat models revealed that (*S*,*S*)-2 displays, after intraperitoneal injection, higher analgesic effects in paw pressure test during acute pain and superior anti-opioid effects in stress-induced analgesia compared to PLG 1 (Fig. 1).^11*a*,*b*^ Because PLG 1 is known to cross the blood–brain barrier (BBB),^14^ we hypothesized that the superior analgesic effect of (*S*,*S*)-2 could be probably due to the presence of the CF_3_ group. Indeed, the significant decrease of the proline basicity^7^ and its low protonation ability should lead to an increase in the hydrophobicity parameter enabling the fluorinated PLG analogue (*S*,*S*)-2 to better cross the BBB and enter the CNS. In order to confirm our hypothesis, we report here the synthesis of four CF_3_-proline analogues of PLG. Their hydrophobicity as well as their ability to cross the BBB were assessed and compared to PLG 1.

## Results and discussion

### Synthesis of the CF_3_-proline analogues of PLG

Four fluorinated analogues of PLG, corresponding to two pairs of diastereomers, have been considered to evaluate the effect of the CF_3_ group in α position of the N-terminal amino group on hydrophobicity and membrane permeation. The collected data should also provide valuable information related to the influence of the configuration of the chiral center bearing the CF_3_ group and its position along the 5-membered ring. In addition to the (*S*)-α-CF_3_-proline (*S*)-4 used for the synthesis of peptide (*S*,*S*)-2, three other fluorinated amino acids, namely the (*R*)-α-CF_3_-proline (*R*)-4, the (*S*,*S*)-CF_3_-pseudoproline (*S*,*S*)-5 and the (*R*,*S*)-CF_3_-pseudoproline (*R*,*S*)-5, were selected to substitute the proline residue into the PLG sequence. The preparation of these trifluoromethylated amino acids in enantiopure form has been previously reported by our group.^8*a*,*d*^

**Scheme 1:**
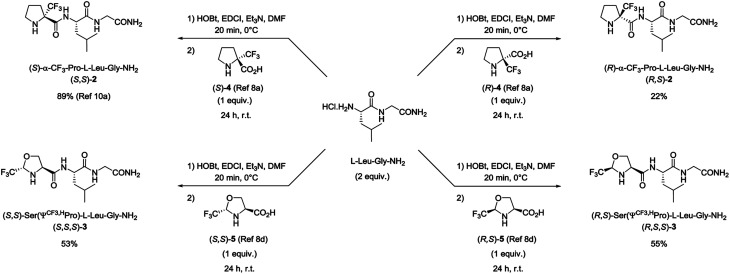
Synthesis of CF_3_-proline and CF_3_-pseudoproline containing PLG analogues.

The synthesis of the corresponding peptides [(*R*,*S*)-2, (*S*,*S*,*S*)-3 and (*R*,*S*,*S*)-3] has been performed following the protocol described for the synthesis of (*S*,*S*)-2.^11*a*^ As mentioned before, the incorporation of the fluorinated proline surrogates into the peptide chain does not required preliminary protection of the amino group. Nevertheless, the reverse addition of the unprotected CF_3_-amino acids to the preformed hydrochloride salt of (*S*)-Leu-Gly-NH_2_ dipeptide using the classical 1-(3-dimethylaminopropyl)-3-ethylcarbodiimide hydrochloride (EDCI) and 1-hydroxybenzotriazole (HOBt) coupling reagents is required to prevent the formation of diketopiperazine side products. While the synthesis of the (*S*)-α-CF_3_-proline containing peptide (*S*,*S*)-2 occurred in very good yield (89%), the coupling reaction starting from (*R*)-α-CF_3_-proline (*R*)-4 gave the corresponding peptide (*R*,*S*)-2 in low yield (22%) (Scheme 1). This result may be explained by a mismatched double stereodifferentiation between the (*R*)-α-CF_3_-proline residue and the (*S*)-Leu-Gly-NH_2_ dipeptide. The peptide 3 diastereomers [(*S*,*S*,*S*)-3 and (*R*,*S*,*S*)-3] were obtained by coupling (*S*,*S*)- and (*R*,*S*)-CF_3_-pseudoproline 5 with the (*S*)-Leu-Gly-NH_2_ dipeptide (53% and 55% respectively).

### Measurement of the isocratic hydrophobicity index *φ*_0_

The hydrophobicity of the fluorinated analogues of PLG has then been assessed and compared to the native PLG 1 in order to investigate the specific effect imparted by the CF_3_ group. For this purpose, we applied a chromatographic method, first developed by Valko *et al.*,^15^ which proved to be very convenient for evaluating the hydrophobicity index of fluorinated peptides.^16^ It is based on the measurement of the isocratic hydrophobicity index (*φ*_0_) which is defined as the percentage (by volume) of organic solvent required to achieve an equal distribution of compound between the polar mobile and apolar stationary phases. Thus, higher is the *φ*_0_ value, more hydrophobic is the compound. The method involves the measurement of the logarithm of retention factor *k* (log *k*) values by reversed-phase high-performance liquid chromatography (RP-HPLC) using various organic-phase concentrations in the mobile phase (in our case, the acetonitrile is used). By plotting the log *k* values as a function of the acetonitrile concentration, the *φ*_0_ value can be obtained from the slope and the intercept of the linear regression (*φ*_0_ = −intercept/slope). A good correlation between the *φ*_0_ and the log *P* values was shown.^15^ The hydrophobicity index of our peptides was first measured at pH 7. The fluorinated PLG analogues 2 and 3 (entries 2–5, Table 1) display similar *φ*_0_ values (*ca.* 30) which are significantly higher (by a factor ∼2.5) compared to the native PLG 1 (entry 1, Table 1). These results show that the incorporation of a CF_3_ group in α position of the N-terminal amino group enhances the hydrophobic character of the corresponding peptide. However, the configuration of the stereocenter bearing the CF_3_ group as well as its position along the pyrrolidine ring does not seem to play a significant role (entries 2–5, Table 1). The *φ*_0_ values were then measured at pH 2 to investigate the impact of the possible ionic form of the PLG peptides 1–3 on the hydrophobicity parameter. The lower *φ*_0_ value observed for the native PLG 1 is consistent with its expected polar ionic state (entry 1, Table 1). Surprisingly, the fluorinated PLG analogues 2 and 3 display a different behaviour at pH 2. The PLG analogues 2 bearing an α-CF_3_-proline exhibit significant decrease of their hydrophobicity index (entries 2 and 3, Table 1), the (*R*,*S*)-2 derivative being more sensitive than its diastereomer (*S*,*S*)-2. These results suggest that the protonation of the deactivated N-terminal amino group occurs at pH 2, increasing the polarity of the corresponding ionized peptides. In contrast, the pseudoproline containing derivatives 3 do not seem to be protonated at pH 2. Only a slight decrease in the *φ*_0_ value is observed for the (*S*,*S*,*S*)-3 (entry 4, Table 1), that of the (*R*,*S*,*S*)-3 diastereomer remaining almost unchanged (entry 5, Table 1). Nevertheless, the incorporation of a Tfm-AA residue at the N-terminal position of the PLG allows to significantly enhance the hydrophobic character.

**Table 1:** *φ*_0_ values for PLG 1 and its fluorinated analogues 2 and 3

Entry	Compound	*φ*_0_ a	
		pH 2	pH 7
1	1	9,2	12,3
2	(*S*,*S*)-2	19,9	31,3
3	(*R*,*S*)-2	17,9	31,8
4	(*S*,*S*,*S*)-3	25,3	27,5
5	(*R*,*S*,*S*)-3	30,4	30,8

a Parameter definition and its calculation is provided in Experimental section.

### Evaluation of the passive diffusion transport by parallel artificial membrane permeability assay (PAMPA)

We then decided to evaluate the ability of our fluorinated PLG analogues 2 and 3 to cross the BBB through passive diffusion. Passive diffusion is a nonsaturable and spontaneous transport process that depends on physicochemical properties such as hydrophobicity, hydrogen bonding, p*K*_a_ or molecular weight. Therefore, such evaluation should allow us to assess the relevance of the CF_3_ group in the passive diffusion transport and thus, demonstrate the benefit in incorporating Tfm-AAs at the N-terminus of peptides. For this purpose, we used PAMPA method, first introduced by Kansy *et al.*,^17^ and selected a mixture of porcine brain polar lipid extract as *in vitro* model of the BBB.^18^ It consists in measuring the ratio of peptide that has been transferred from a donor well into an acceptor well through the artificial membrane (see Experimental part). The rate of permeation can be determined by the effective permeability (*P*_e_) as well as the rate of transport values. The membrane retention value can also be obtained and reflects the strength of the interaction between the lipids that mimic the membrane and the peptide. While PLG 1 is known to cross the BBB,^13^ its permeability was found to be very negligible and no peptide was retained in the membrane (entry 1, Table 2). Thus, PLG 1 seems to not display excellent physicochemical properties to transport through passive diffusion. This result correlates with the fact that PLG 1 has been reported to enter brain through a partially saturable transport system.^14^ PLG analogues 2, bearing the α-CF_3_-proline residue, show an ability to cross the membrane (entries 2 and 3, Table 2). According to the empirical BBB permeation prediction,^19^ the peptide (*R*,*S*)-2 displays a poor effective permeability (*P*_e_ < 2.0 × 10^6^ cm s^−1^) while its diastereomer (*S*,*S*)-2 exhibits a moderate transport properties (2.0 × 10^6^ < *P*_e_ < 4.0 × 10^6^ cm s^−1^). Both peptides (*S*,*S*)-2 and (*R*,*S*)-2 were also found to have a strong interaction with the membrane. It is worth noting the relevant difference in terms of permeability due to the change of only one stereocenter in the molecules. On the contrary, none of the CF_3_-pseudoproline containing PLG analogues 3 reveal an ability to cross the membrane by passive diffusion nor a membrane affinity (entries 4 and 5, Table 2). The PAMPA assays have been performed at pH 7.4, a pH for which fluorinated PLG analogues 2 and 3 display similar hydrophobicity index values (see Table 1). Therefore, the significant difference between the peptides 2 and 3 suggests that the nature of the 5-membered ring (pyrrolidine or oxazolidine) and the position along the ring as well as the absolute configuration of the carbon bearing the CF_3_ group play also a key role in the passive diffusion transport.

**Table 2:** Effective permeability (*P*_e_), percentage of transport and membrane retention after 4 h in the PAMPA of PLG 1 and its fluorinated analogues 2 and 3^a^

Entry	Compound	*P*_e_ (× 10^6^)^b^ cm s^−1^	Transport (%) (4 h)^b^	Membrane retention^b^
1	1	nd^c^	nd^c^	nd^c^
2	(*S*,*S*)-2	2.68 ± 0.04	5.34 ± 0.08	50%
3	(*R*,*S*)-2	0.31 ± 0.03	0.65 ± 0.06	30%
4	(*S*,*S*,*S*)-3	nd^c^	nd^c^	nd^c^
5	(*R*,*S*,*S*)-3	nd^c^	nd^c^	nd^c^

a Data are expressed as the mean ± SD.

b Parameters definitions and their calculations are provided in Experimental section.

c Not determined.

## Conclusions

We demonstrate that the incorporation of a Tfm-AA residue at the N-terminal position of a peptide can be very useful to enhance its hydrophobic character. Moreover, depending on its position along the 5-membered pyrrolidine ring, the CF_3_ group may promote passive diffusion transport. Thus, Tfm-AA residues could serve as potent BBB-shuttle to transport into the CNS compounds of interests that cannot cross the BBB. The use of peptide as BBB-shuttles is one of the most promising approaches to deliver safely drugs to the brain.^20^

## Experimental section

### Synthesis of the CF_3_-Proline analogues of PLG

#### Representative procedure for the peptide coupling reaction

To a solution of l-Leu-Gly-NH_2_ hydrochloride salt (2 equiv.) in DMF were successively added at 0 °C NEt_3_ (4.1 equiv.), HOBt (1.5 equiv.) and EDCI (1.5 equiv.). The reaction mixture was stirred for 20 min at 0 °C and the CF_3_-proline surrogate (*R*)-4, (*R*,*S*)-5 or (*S*,*S*)-5 (1 equiv.) was added. After 20 min at 0 °C, the resulting solution was stirred overnight at room temperature and then diluted with CH_2_Cl_2_ and water. The aqueous layer was extracted with CH_2_Cl_2_ (3×) and the combined chlorinated extracts were washed with water, dried with MgSO_4_, filtered and concentrated under reduced pressure. Purification by flash chromatography gave fluorinated peptides (*R*,*S*)-2, (*S*,*S*,*S*)-3 and (*R*,*S*,*S*)-3 in 22–53% yield.

#### (*R*)-*α*-CF_3_-Pro-l-Leu-Gly-NH_2_ (*R*,*S*)-2

The tripeptide (*R*,*S*)-2 was prepared according to the representative procedure, with HCl.l-Leu-Gly-NH_2_ (610 mg, 2.73 mmol, 2 equiv.), NEt_3_ (844 μL, 5.60 mmol, 4.1 equiv.), HOBt (276 mg, 2.05 mmol, 1.5 equiv.), EDCI (391 mg, 2.05 mmol, 1.5 equiv.), and (*R*)-α-CF_3_-proline (*R*)-4 (250 mg, 1.37 mmol, 1 equiv.) in DMF (6 mL). Purification on silica gel (CH_2_Cl_2_/MeOH, 90 : 10) gave pure (*R*,*S*)-2 (110 mg, 22%) as colorless oil. *R*_f_ = 0.33 (CH_2_Cl_2_/MeOH, 90 : 10); [α]_D_ +20.1 (*c* 1.4 in MeOH). IR (neat): 3272, 2955, 1651, 1651, 1519, 1386, 1250 cm^−1^; ^1^H NMR (400 MHz, CD_3_OD) *δ* 0.93 (d, *J* = 6.4 Hz, 3H, H_*δ*_ Leu-H), 0.96 (d, *J* = 6.4 Hz, 3H, H_*δ*_ Leu-H), 1.58–1.72 (m, 3H, H_*β*_ Leu-H, H_*γ*_ Leu-H), 1.73–1.90 (m, 2H, H_*γ*_ Pro-H), 2.12–2.28 (m, 2H, H_*β*_ Pro), 3.02–3.14 (m, 2H, H_*δ*_ Pro), 3.78 (d, *J* = 17.0 Hz, 1H, H_*α*_ Gly-Ha), 3.91 (d, *J* = 17.0 Hz, 1H, H_*α*_ Gly-Hb), 4.41 (m, 1H, H_*α*_ Leu-H); ^13^C NMR (100.5 MHz, CD_3_OD) *δ* 22.0 (CH_3_, C_*δ*_ Leu), 23.4 (CH_3_, C_*δ*_ Leu), 25.9 (CH, C_*γ*_ Leu), 26.1 (CH_2_, C_*γ*_ Pro), 33.2 (CH_2_, C_*β*_ Pro), 41.8 (CH_2_, C_*β*_ Leu), 43.0 (CH_2_, C_*α*_ Gly), 48.2 (CH_2_, C_*δ*_ Pro), 53.5 (CH, C_*α*_ Leu), 72.2 (q, *J* = 25.9 Hz, C, C_*α*_ Pro), 127.4 (q, *J* = 283.7 Hz, C, CF_3_), 172.1 (C, C=O), 173.9 (C, C=O), 174.6 (C, C=O); ^19^F NMR (376.2 MHz, CD_3_OD): *δ* −78.9 (s, CF_3_); HRMS (ESI-TOF) calcd. for C_14_H_24_N_4_O_3_F_3_ [M + H]^+^ 353.1801, found 353.1801.

#### (2*R*,4*S*)-Ser(Ψ^CF^3^,H^Pro)-l-Leu-Gly-NH_2_ (*R*,*S*,*S*)-3

The tripeptide (*R*,*S*,*S*)-3 was prepared according to the representative procedure, with HCl.l-Leu-Gly-NH_2_ (364 mg, 1.63 mmol, 1.5 equiv.), NEt_3_ (690 μL, 4.34 mmol, 4 equiv.), HOBt (220 mg, 1.63 mmol, 1.5 equiv.), EDCI (311 mg, 1.63 mmol, 1.5 equiv.) and (2*R*,4*S*)-Ser(Ψ^CF^3^,H^Pro) (*R*,*S*)-5 (200 mg, 1.08 mmol, 1 equiv.) in DMF (6 mL). Purification on silica gel (CH_2_Cl_2_/MeOH, 90 : 10) gave pure (*R*,*S*,*S*)-3 (212 mg, 55%) as hygroscopic white solid. *R*_f_ = 0.34 (CH_2_Cl_2_/MeOH, 90 : 10); [α]_D_ −12.2 (*c* 1.0 in MeOH); IR (neat): 3326, 2961, 2470, 1638, 1524, 1454, 1288 cm^−1^; ^1^H NMR (400 MHz, CD_3_OD) *δ* 0.93 (d, *J* = 6.4 Hz, 3H, H_*δ*_ Leu-H), 0.96 (d, *J* = 6.0 Hz, 3H, H_*δ*_ Leu-H), 1.58–1.70 (m, 3H, H_*β*_ Leu-H and H_*γ*_ Leu-H), 3.77 (d, *J* = 16.9 Hz, 1H, H_*α*_ Gly-Ha), 3.90 (d, *J* = 16.9 Hz, 1H, H_*α*_ Gly-Hb), 4.11–4.21 (m, 3H, H_*α*_ Ψpro-H and H_*β*_ Ψpro-Ha), 4.37 (t, *J* = 6.5 Hz, 1H, H_*α*_ Leu-H), 5.10 (d, *J* = 6.0 Hz, 1H, H_*δ*_ Ψpro-H); ^13^C NMR (100.5 MHz, CD_3_OD) *δ* 21.7 (CH_3_, C_*δ*_ Leu), 23.5 (CH_3_, C_*δ*_ Leu), 25.5 (CH, C_*γ*_ Leu), 42.0 (CH_2_, C_*β*_ Leu), 43.1 (CH_2_, C_*α*_ Gly), 53.1 (CH, C_*α*_ Leu), 60.8 (CH, C_*α*_ Ψpro), 70.6 (CH_2_, C_*β*_ Ψpro), 89.1 (q, *J* = 33.6 Hz, CH, C_*δ*_ Ψpro), 125.1 (q, *J* = 282.8 Hz, C, CF_3_), 174.2 (C, C=O), 174.6 (C, C=O), 175.0 (C, C=O); ^19^F NMR (376.2 MHz, CD_3_OD) *δ* −85.3 (bs, CF_3_); HRMS (ESI-TOF) calcd. for C_13_H_22_N_4_O_4_F_3_ [M + H]^+^ 355.1593, found 355.1601.

#### (2*S*,4*S*)-Ser(Ψ^CF^3^,H^Pro)-l-Leu-Gly-NH_2_ (*S*,*S*,*S*)-3

The tripeptide (*S*,*S*,*S*)-3 was prepared according to the representative procedure, with HCl.l-Leu-Gly-NH_2_ (1.11 g, 4.63 mmol, 2 equiv.), NEt_3_ (1.5 mL, 9.50 mmol, 4.1 equiv.), HOBt (469 mg, 3.47 mmol, 1.5 equiv.), EDCI (664 mg, 3.47 mmol, 1.5 equiv.), and (2*S*,4*S*)-Ser(Ψ^CF^3^,H^Pro) (*S*,*S*)-5 (426 mg, 2.32 mmol, 1 equiv.) in DMF (10 mL). Purification on silica gel (CH_2_Cl_2_/MeOH, 90 : 10) gave pure (*S*,*S*,*S*)-3 (434 mg, 53%) as white solid. *R*_f_ = 0.34 (CH_2_Cl_2_/MeOH, 90 : 10); mp 150 °C; [α]_D_ −41.8 (*c* 0.9 in MeOH); IR (neat): 3301, 2419, 1782, 1558, 1435, 1289 cm^−1^; ^1^H NMR (400 MHz, CD_3_OD) *δ* 0.95 (d, *J* = 6.4 Hz, 3H, H_*δ*_ Leu-H), 0.99 (d, *J* = 6.4 Hz, 3H, H_*δ*_ Leu-H), 1.58–1.64 (m, 2H, H_*β*_ Leu-H), 1.68 (m, 1H, H_*γ*_ Leu-H), 3.64 (t, *J* = 7.3 Hz, 1H, H_β_ Ψpro-Ha), 3.74 (d, *J* = 17.4 Hz, 1H, H_*α*_ Gly-Ha), 3.92 (d, *J* = 17.4 Hz, 1H, H_*α*_ Gly-Hb), 3.98 (t, *J* = 7.3 Hz, 1H, H_*α*_ Ψpro-H), 4.21 (t, *J* = 7.3 Hz, 1H, H_β_ Ψpro-Hb), 4.35 (t, *J* = 6.9 Hz, 1H, H_*α*_ Leu-H), 5.14 (q, *J* = 5.5 Hz, 1H, H_*δ*_ Ψpro-H); ^13^C NMR (100.5 MHz, CD_3_OD) *δ* 22.0 (CH_3_, C_*δ*_ Leu), 23.3 (CH_3_, C_*δ*_ Leu), 25.9 (CH, C_*γ*_ Leu), 41.4 (CH_2_, C_*β*_ Leu), 43.1 (CH_2_, C_*α*_ Gly), 54.0 (CH, C_*α*_ Leu), 60.2 (CH, C_*α*_ Ψpro), 71.4 (CH_2_, C_*β*_ Ψpro), 89.4 (q, *J* = 33.6 Hz, CH, C_*δ*_ Ψpro), 125.0 (q, *J* = 282.8 Hz, C, CF_3_), 172.3 (C, C=O), 174.2 (C, C=O), 175.0 (C, C=O); ^19^F NMR (376.2 MHz, CD_3_OD) *δ* −86.1 (d, *J* = 5.5 Hz, CF_3_); HRMS (ESI-TOF) calcd. for C_13_H_22_N_4_O_4_F_3_ [M + H]^+^ 355.1593, found 355.1587.

### Isocratic hydrophobicity index *φ*_0_

An Agilent 1200 series high-performance liquid chromatograph coupled to an ELSD detector was used. The reversed-phase HPLC measurements were carried out on a Zorbax® RX-C18 analytical column (4.6 × 250 mm, 5 μm). The mobile phase was 50 mM ammonium acetate and HPLC grade acetonitrile as organic modifier (pH 7.0) or TFA 0.1% buffer aqueous solution and 0.1% TFA HPLC grade acetonitrile (pH 2). The mobile phase flow rate was ranging from 0.1–1.0 mL min^−1^. The dead time (*t*_0_) was measured by injecting sodium nitrate together with the sample at a concentration of 10 mM. The samples were dissolved at 1 mg/1 mL in water. Few drops of acetonitrile were added when the dissolution of the peptide was incomplete. The logarithm of retention factor *k* (log *k*) values were obtained according to the eqn (1):1
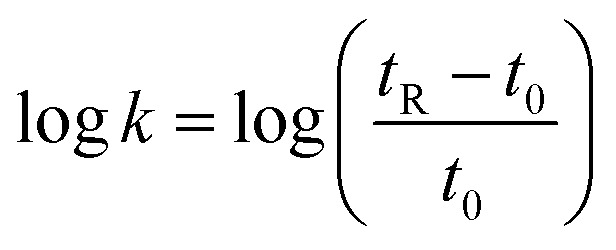
where *t*_R_ is the retention time of the compound and *t*_0_ the retention time of the unretained sodium nitrate solute.

The log *k* values were plotted as a function of the acetonitrile concentration. The slope (*S*) and the intercept (log *k*_w_) values were calculated from at least three concentrations of acetonitrile. The correlation coefficients of the linear fit were higher than 0.99. The isocratic hydrophobicity index *φ*_0_ was calculated according to the eqn (2):

2 *φ*_0_ = −log *k*_w_/*S*

where log *k*_w_ is the log *k* value extrapolated to the 0% acetonitrile concentration.

### Parallel artificial membrane permeability assay (PAMPA)

The PAMPA was used to measure the capacity of the peptides 1–3 to cross the BBB by passive diffusion. The effective permeability (*P*_e_) of the compounds was determined at an initial concentration of 5 mM. The buffer solution was prepared from a commercially concentrated one, supplied by pION. Following the manufacturer's instructions, system solution was adjusted to pH 7.4 using a 0.5 M NaOH solution. The compounds to be analyzed were dissolved in a mixture of buffer solution with 20% of 1-propanol used as co-solvent to ensure their solubility. The PAMPA sandwich was separated, and the donor wells were filled with 195 μL of each compound. A magnetic stirrer was placed in each well. The acceptor plate was put into the donor plate, ensuring that the underside of the membrane was in contact with the buffer. Then, 4 μL of a mixture of phospholipids from a porcine polar brain extract (20 mg mL^−1^) in dodecane (composition: 12.6% phosphatidylcholine (PC), 33.1% phosphatidylethanolamine (PE), 18.5% phosphatidylserine (PS), 4.1% phosphatidylinositol (PI), 0.8% phosphatidic acid and 30.9% of other compounds) was added to the filter of each well, followed by 200 μL of buffer solution. The plate was then covered and incubated at room temperature in a saturated humidity atmosphere for 4 h under orbital agitation at 25 μm of unstirred water layer (UWL). After this period, the solution from the donor and acceptor plates was transferred to the UPLC vials. 100 μL of each acceptor and donor samples and 100 μL of *t*_0_ samples were injected into the UPLC apparatus. Acceptor samples were analyzed by MALDI-TOF in order to study the presence of not degraded peptides and confirm their crossing. The effective permeability (*P*_e_) after 4 h was calculated using eqn (3) and the percentage of transport with eqn (4):3
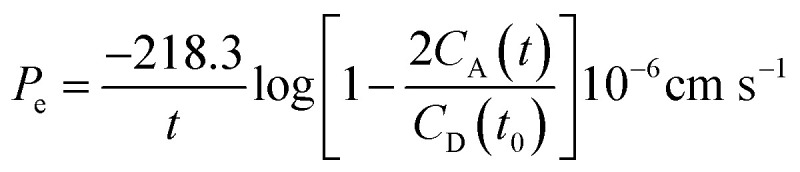
4
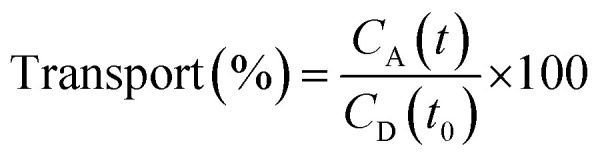
where *t* is time (h), *C*_A_ (*t*) is the peptide concentration in the acceptor well at time *t*, and *C*_D_ (*t*_0_) is the peptide concentration in the donor well at 0 h.

Membrane retention was also calculated as the difference of product at *t*_0_ and after 4 h in the donor and acceptor compartments.

## Conflicts of interest

There are no conflicts to declare.

## Supplementary Material

RA-008-C8RA02511H-s001
