# Supplementary material for: Trifluoromethylated proline analogues as efficient tools to enhance the hydrophobicity and to promote passive diffusion transport of the l-prolyl-l-leucyl glycinamide (PLG) tripeptide
Source: RSC Adv. 2018 Apr 18;8(26):14597–602. doi: 10.1039/c8ra02511h (PMC9079923; doi:10.1039/c8ra02511h)

Electronic Supplementary Material (ESI) for RSC Advances.
This journal is © The Royal Society of Chemistry 2018

Electronic Supplementary Information (ESI)

Trifluoromethylated-proline analogues as efficient tool to enhance the hydrophobicity and to promote passive diffusion transport of the L-prolyl-L-leucylglycinamide (PLG) tripeptide

Martin Oliver,[a] Charlène Gadais,[a] Júlia García-Pindado,[b] Meritxell Teixidó,[b] Nathalie Lensen,*[a] Grégory Chaume*[a] and Thierry Brigaud[a]

[a]Laboratoire de Chimie Biologique (LCB), Université de Cergy-Pontoise, 5 mail Gay-Lussac, Neuville-sur-Oise, 95031 Cergy-Pontoise, France. E-mail: nathalie.lensen@u-cergy.fr, gregory.chaume@u-cergy.fr

[b]Institute for Research in Biomedicine (IRB Barcelona), Barcelona Institute of Science and Technology (BIST), C/ Baldiri Reixac 10, 08028 Barcelona, Spain.

Table of contents

^{1}H NMR spectrum of (*R*)-α-CF_3-Pro-L-Leu-Gly-NH_2 (*R*,*S*)-2 **S2**

^{13}C NMR spectrum of (*R*)-α-CF_3-Pro-L-Leu-Gly-NH_2 (*R*,*S*)-2 **S3**

^{19}F NMR spectrum of (*R*)-α-CF_3-Pro-L-Leu-Gly-NH_2 (*R*,*S*)-2 **S4**

^{1}H NMR spectrum of (2*R*,4*S*)-Ser($\Psi^{CF3,H}$Pro)-L-Leu-Gly-NH_2 (*R*,*S*,*S*)-3 **S5**

^{13}C NMR spectrum of (2*R*,4*S*)-Ser($\Psi^{CF3,H}$Pro)-L-Leu-Gly-NH_2 (*R*,*S*,*S*)-3 **S6**

^{19}F NMR spectrum of (2*R*,4*S*)-Ser($\Psi^{CF3,H}$Pro)-L-Leu-Gly-NH_2 (*R*,*S*,*S*)-3 **S7**

^{1}H NMR spectrum of (2*S*,4*S*)-Ser($\Psi^{CF3,H}$Pro)-L-Leu-Gly-NH_2 (*S*,*S*,*S*)-3 **S8**

^{13}C NMR spectrum of (2*S*,4*S*)-Ser($\Psi^{CF3,H}$Pro)-L-Leu-Gly-NH_2 (*S*,*S*,*S*)-3 **S9**

^{19}F NMR spectrum of (2*S*,4*S*)-Ser($\Psi^{CF3,H}$Pro)-L-Leu-Gly-NH_2 (*S*,*S*,*S*)-3 **S10**

Determination of φ_0 value for PLG 1 **S11**

Determination of φ_0 value for PLG (*S*,*S*)-2 **S11**

Determination of φ_0 value for PLG (*R*,*S*)-2 **S11**

Determination of φ_0 value for PLG (*S*,*S*,*S*)-3 **S12**

Determination of φ_0 value for PLG (*R*,*S*,*S*)-3 **S12**

Log k values as a function of a percentage of acetonitrile at pH 2 **S13**

Log k values as a function of a percentage of acetonitrile at pH 2 **S14**

^{1}H NMR spectrum of (*R*)-α-CF_3-Pro-L-Leu-Gly-NH_2 (*R*,*S*)-2.

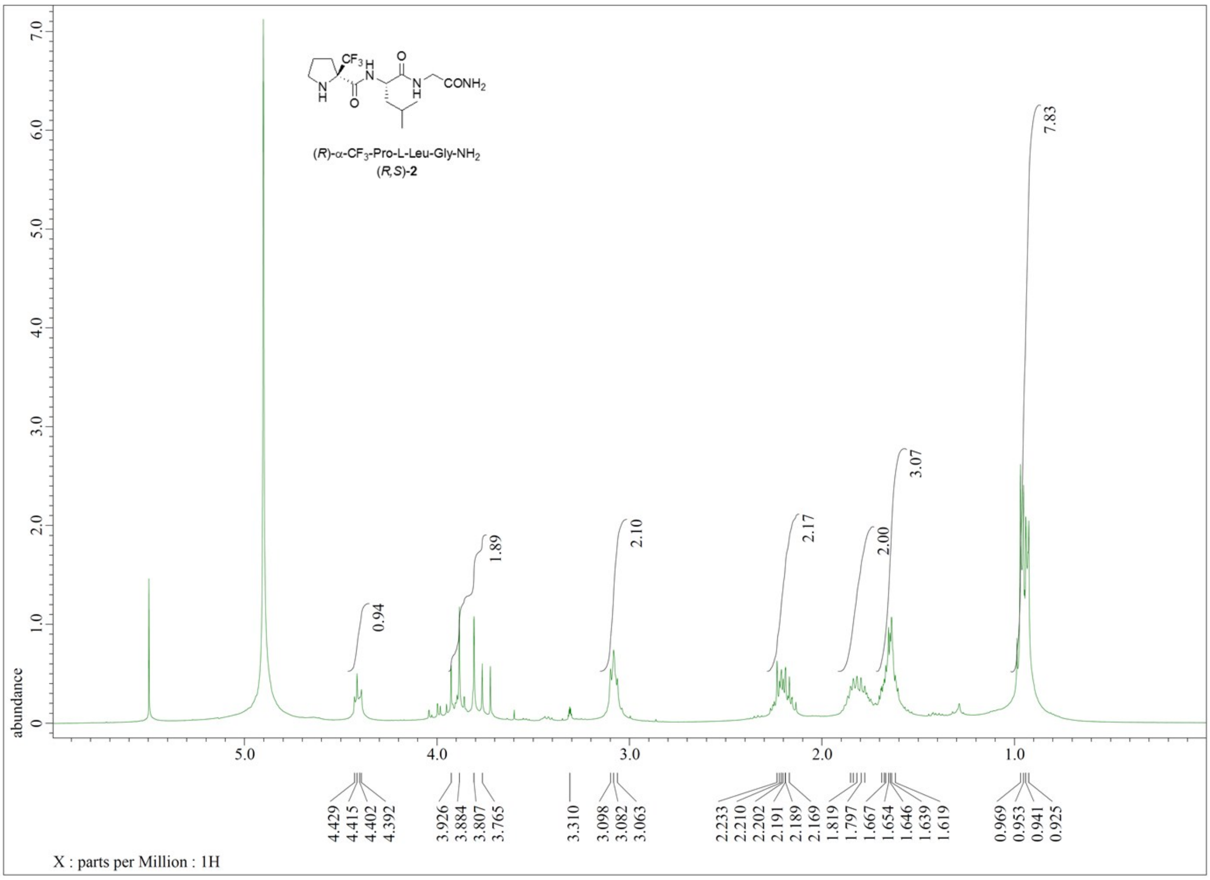

^{13}C NMR spectrum of (*R*)-α-CF_3-Pro-L-Leu-Gly-NH_2 (*R*,*S*)-2.

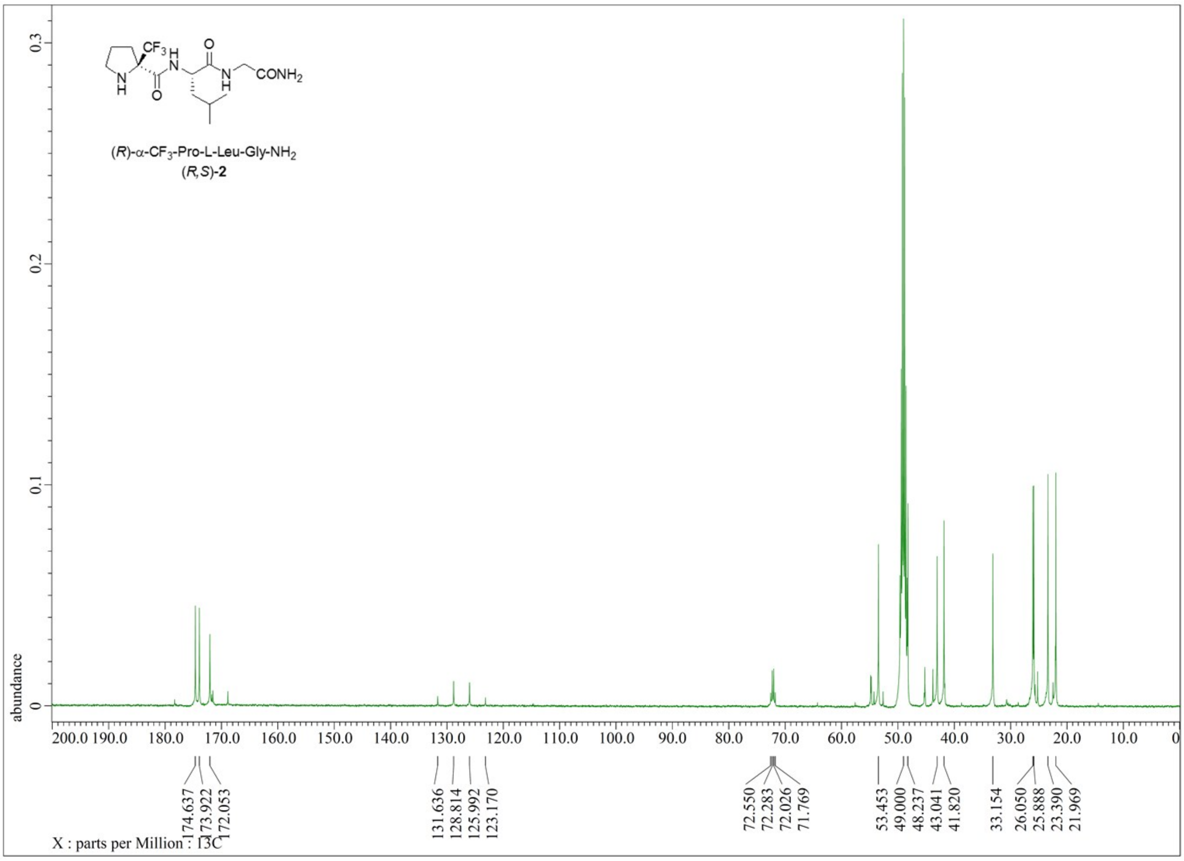

^{19}F NMR spectrum of (*R*)-α-CF_3-Pro-L-Leu-Gly-NH_2 (*R*,*S*)-2.

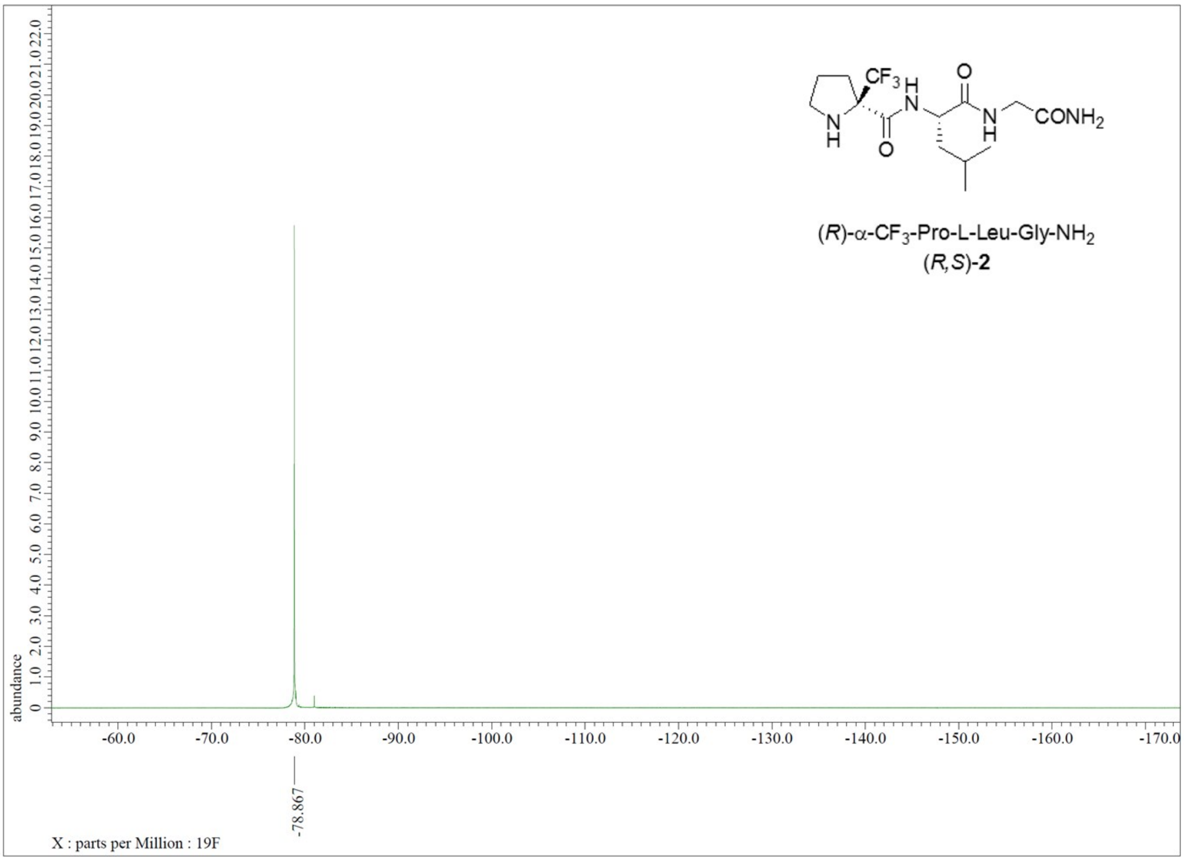

^{1}H NMR spectrum of (2*R*,4*S*)-Ser($\Psi^{CF3,H}$Pro)-L-Leu-Gly-NH$_2$ (*R*,*S*,*S*)-3.

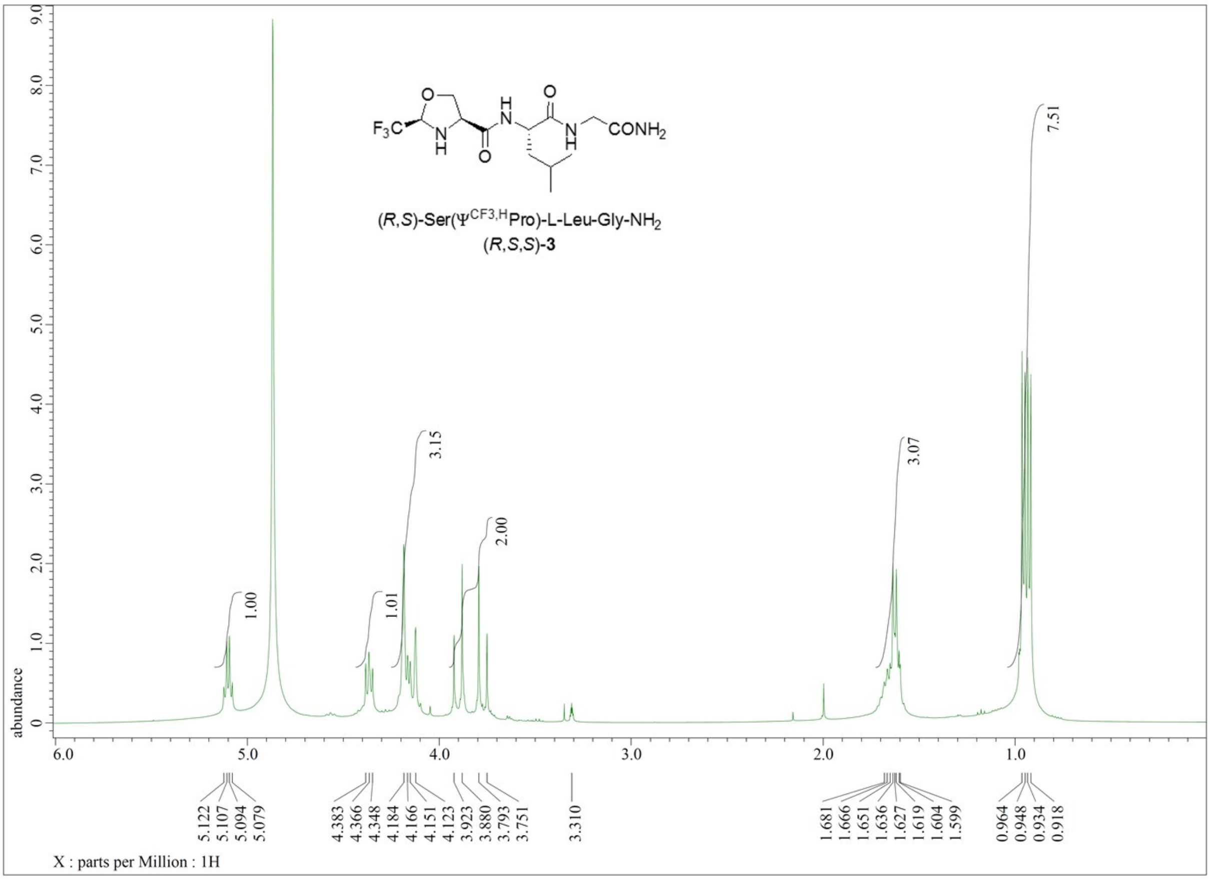

^{13}C NMR spectrum of (2*R*,4*S*)-Ser($\Psi^{CF3,H}$Pro)-L-Leu-Gly-NH$_2$ (*R*,*S*,*S*)-3.

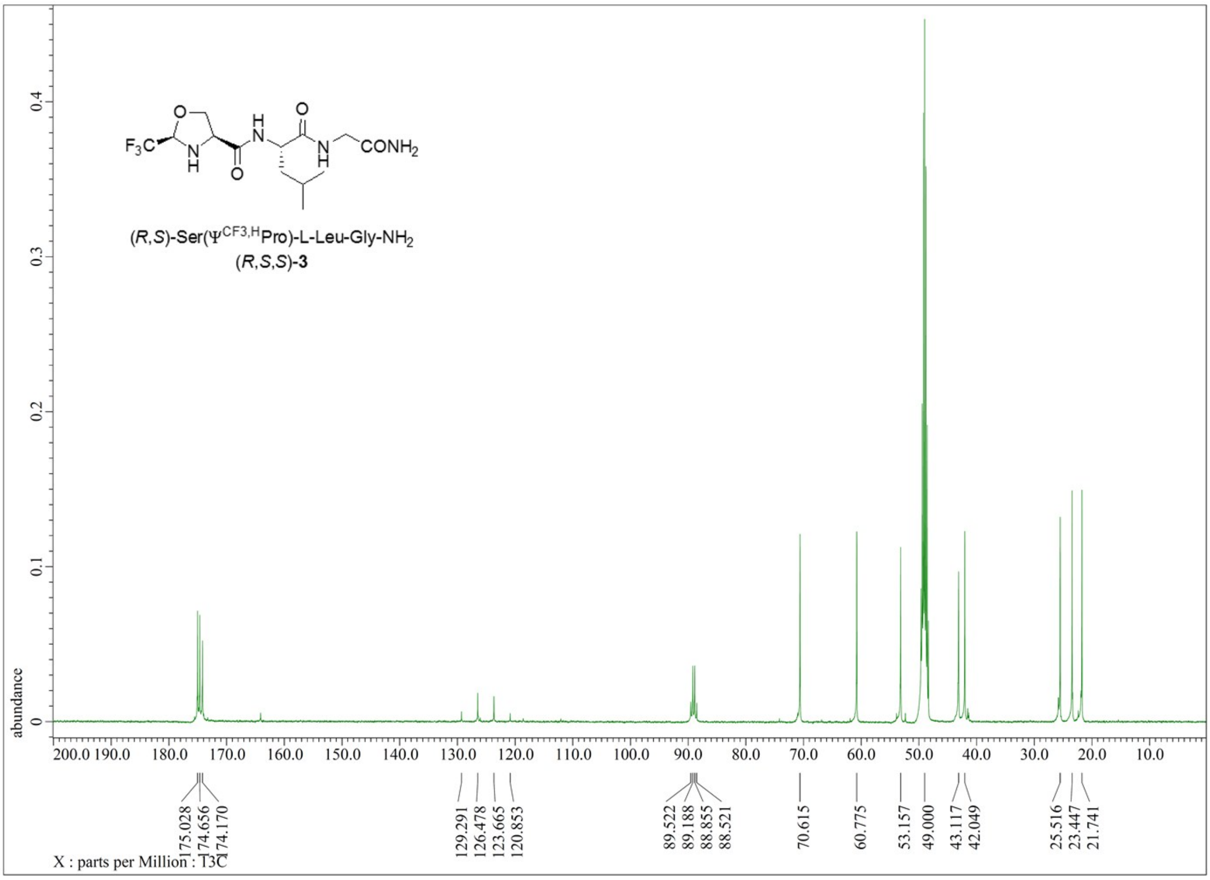

^{19}F NMR spectrum of (2*R*,4*S*)-Ser($\Psi^{CF3,H}$Pro)-L-Leu-Gly-NH$_2$ (*R*,*S*,*S*)-3.

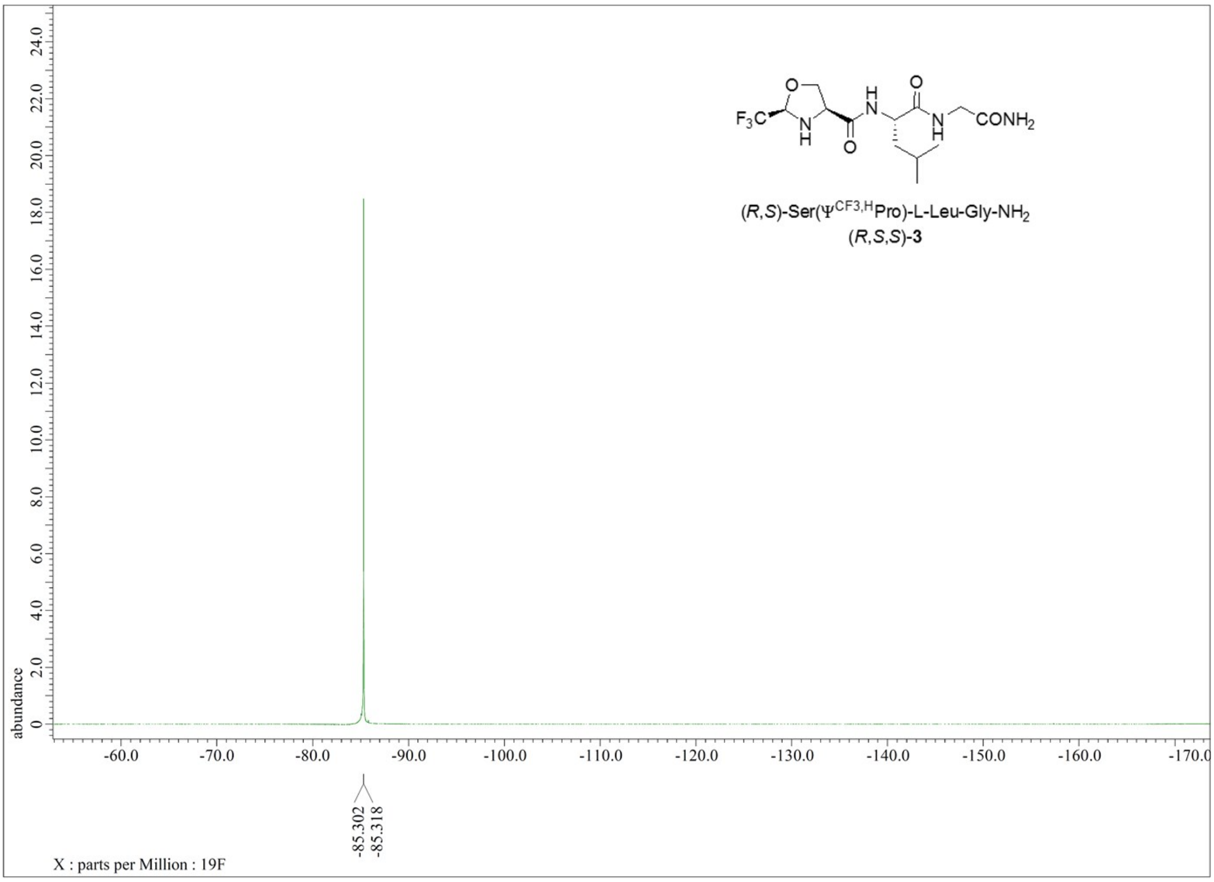

^{1}H NMR spectrum of (2*S*,4*S*)-Ser($\Psi^{CF3,H}$Pro)-L-Leu-Gly-NH$_2$ (*S*,*S*,*S*)-3.

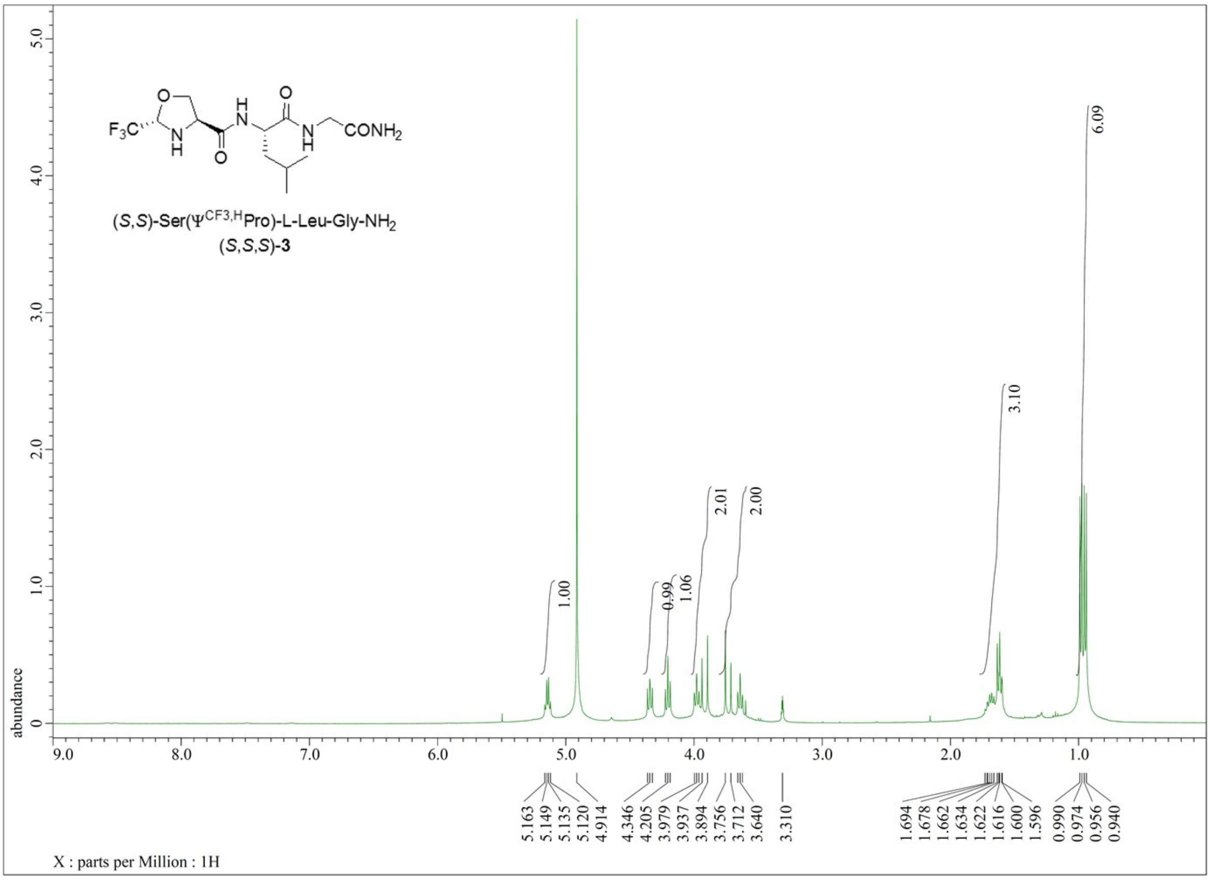

^{13}C NMR spectrum of (2*S*,4*S*)-Ser($\Psi^{CF3,H}$Pro)-L-Leu-Gly-NH$_2$ (*S*,*S*,*S*)-3.

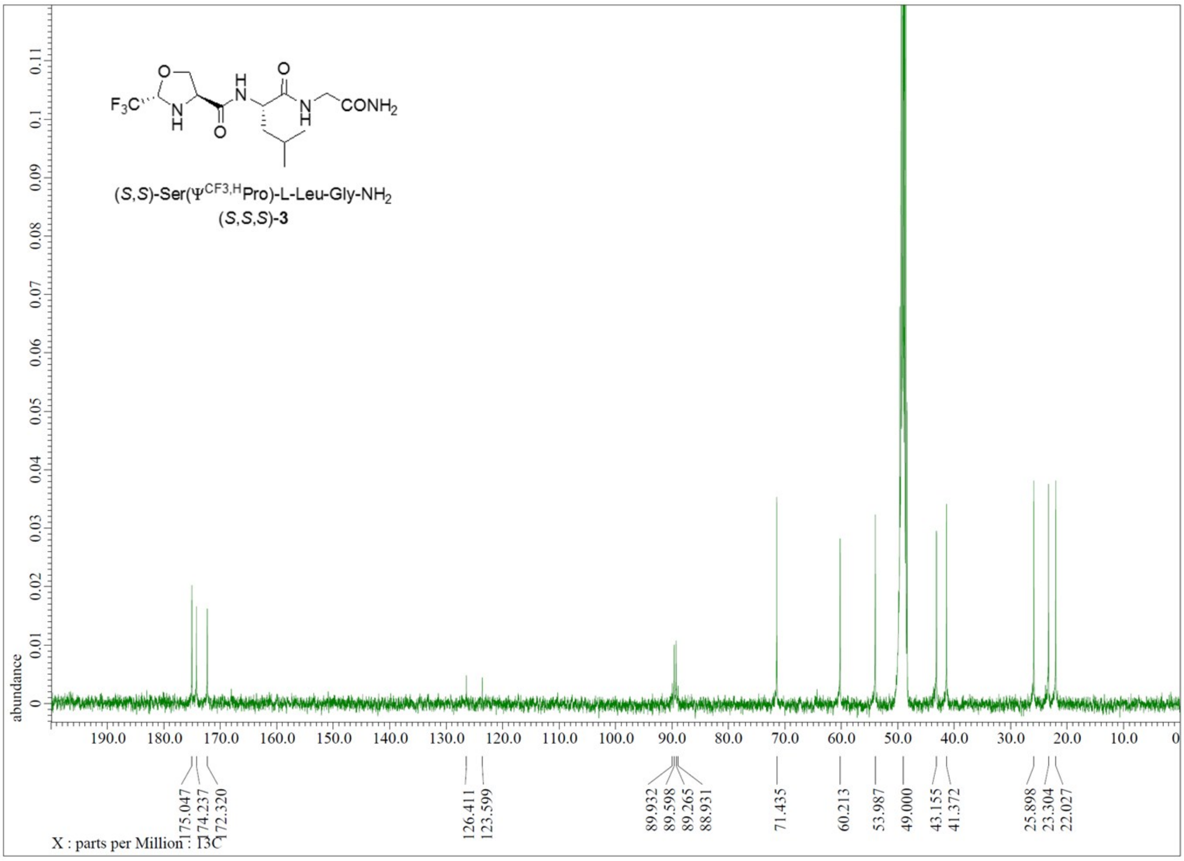

^{19}F NMR spectrum of (2*S*,4*S*)-Ser($\Psi^{CF3,H}$Pro)-L-Leu-Gly-NH$_2$ (*S*,*S*,*S*)-3.

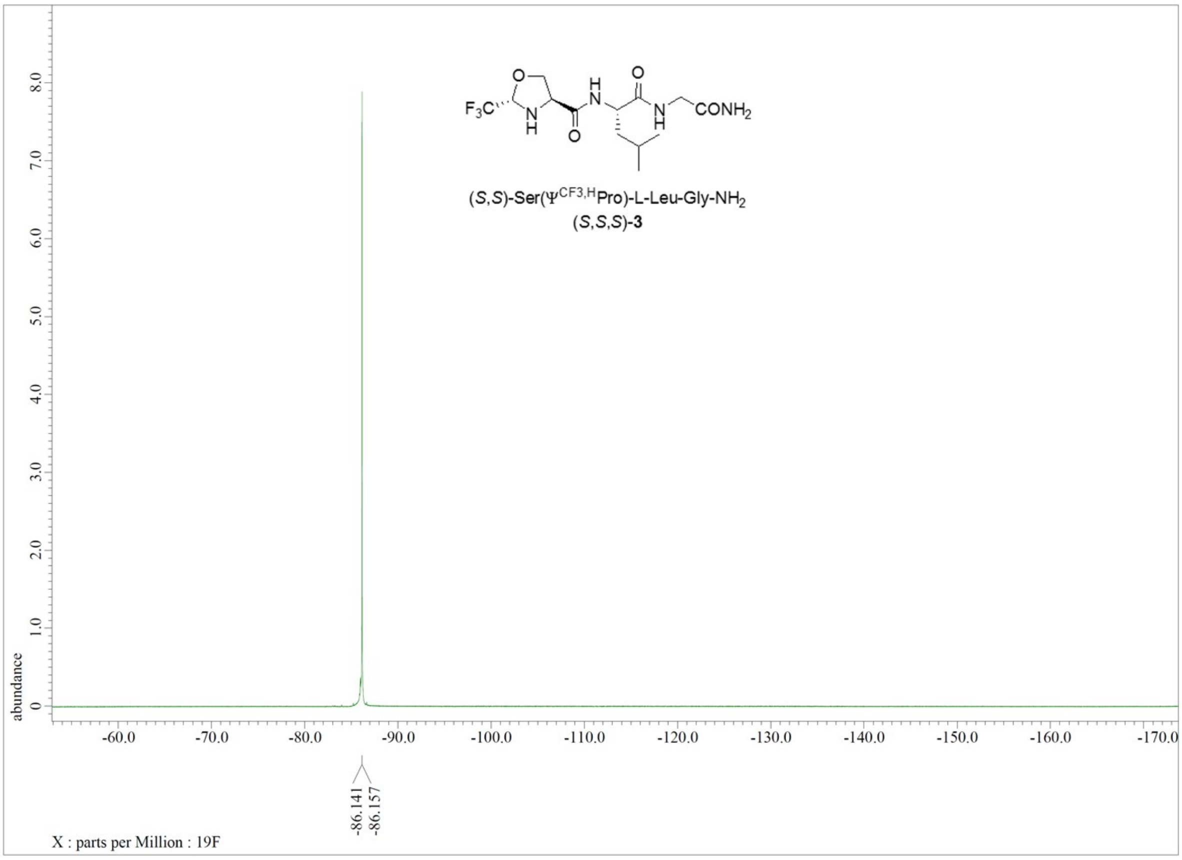

Determination of φ_0 value for PLG 1

	% MeCN	T_R	T_0	k	Log k	φ_0
**pH 2**	8	5.503	2.442	1.2535	0.0981	**9.16**
	10	4.471	2.403	0.8606	-0.0652	
	12	3.845	2.497	0.5398	-0.2677	
	15	2.922	2.298	0.2715	-0.5662	
**pH 7**	8	5.212	2.225	1.3425	0.1279	**12.31**
	12	5.042	2.488	1.0265	0.0114	
	15	4.099	2.242	0.8283	-0.0818	

Determination of φ_0 value for PLG (*S*,*S*)-2

	% MeCN	T_R	T_0	k	Log k	φ_0
**pH 2**	15	7.381	2.367	2.1183	0.3260	**19.90**
	19	4.865	2.329	1.0889	0.0370	
	21	5.142	2.845	0.8074	-0.0929	
	23	6.067	3.697	0.6411	-0.1931	
	25	8.196	5.426	0.5105	-0.2920	
**pH 7**	20	14.218	2.373	4.9916	0.6982	**31.25**
	24	8.881	2.376	2.7378	0.4374	
	26	7.450	2.402	2.1016	0.3225	
	28	6.259	2.398	1.6101	0.2069	
	30	5.420	2.471	1.1934	0.0768	

Determination of φ_0 value for PLG (*R*,*S*)-2

	% MeCN	T_R	T_0	k	Log k	φ_0
**pH 2**	10	11.338	2.399	3.7261	0.5713	**17.92**
	12	8.231	2.221	2.7060	0.4323	
	15	5.825	2.338	1.4914	0.1736	
	18	4.463	2.312	0.9304	-0.0313	
	20	3.906	2.301	0.6975	-0.1564	
	22	3.553	2.278	0.5597	-0.2520	
**pH 7**	20	16.089	2.462	5.5349	0.7431	**31.76**
	24	9.659	2.464	2.9200	0.4654	
	26	8.020	2.469	2.2483	0.3519	
	28	6.690	2.475	1.7030	0.2312	
	30	5.741	2.472	1.3224	0.1214	

Determination of φ_0 value for PLG (*S*,*S*,*S*)-3

	% MeCN	T_R	T_0	k	Log k	φ_0
**pH 2**	20	11.613	2.742	3.2352	0.5099	**25.33**
	22	9.176	2.705	2.3922	0.3788	
	25	6.800	2.667	1.5497	0.1902	
	27	5.824	2.638	1.2077	0.0820	
	40	3.215	2.458	0.3080	-0.5115	
**pH 7**	22	9.339	2.940	2.1765	0.3378	**27.45**
	24	7.531	2.991	1.5179	0.1812	
	26	6.331	2.882	1.1967	0.0780	
	28	5.478	2.841	0.9282	-0.0324	
	30	4.768	2.757	0.7294	-0.1370	

Determination of φ_0 value for PLG (*R*,*S*,*S*)-3

	% MeCN	T_R	T_0	k	Log k	φ_0
**pH 2**	23	11.452	2.856	3.0098	0.4785	**30.35**
	25	8.474	2.745	2.0871	0.3195	
	27	6.891	2.699	1.5532	0.1912	
	29	7.388	3.318	1.2266	0.0887	
	31	10.067	5.206	0.9337	-0.0298	
**pH 7**	23	11.662	2.907	3.0117	0.4788	**30.79**
	24	9.953	2.842	2.5021	0.3983	
	26	8.087	2.788	1.9006	0.2789	
	28	6.509	2.741	1.3747	0.1382	
	30	5.449	2.700	1.0181	0.0078	
	40	3.357	2.565	0.3088	-0.5104	

Log k values as a function of a percentage of acetonitrile at pH 2

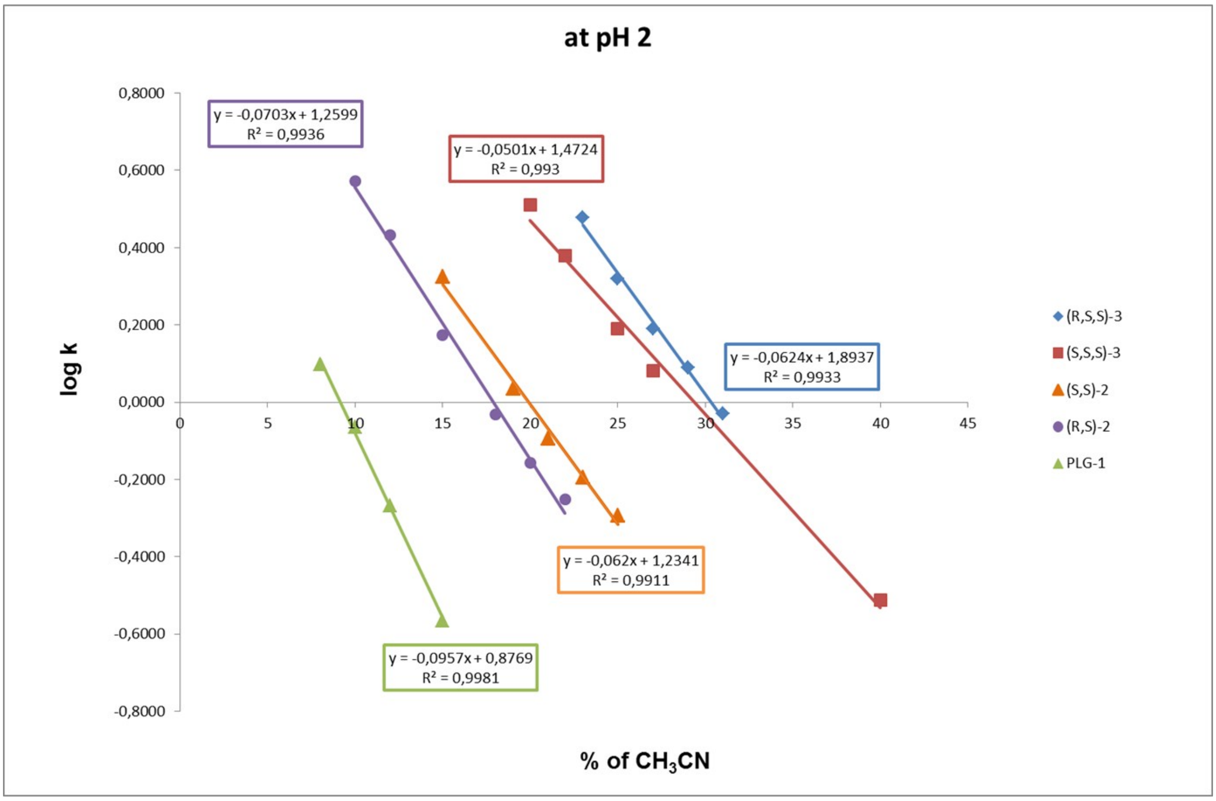

Log k values as a function of a percentage of acetonitrile at pH 7

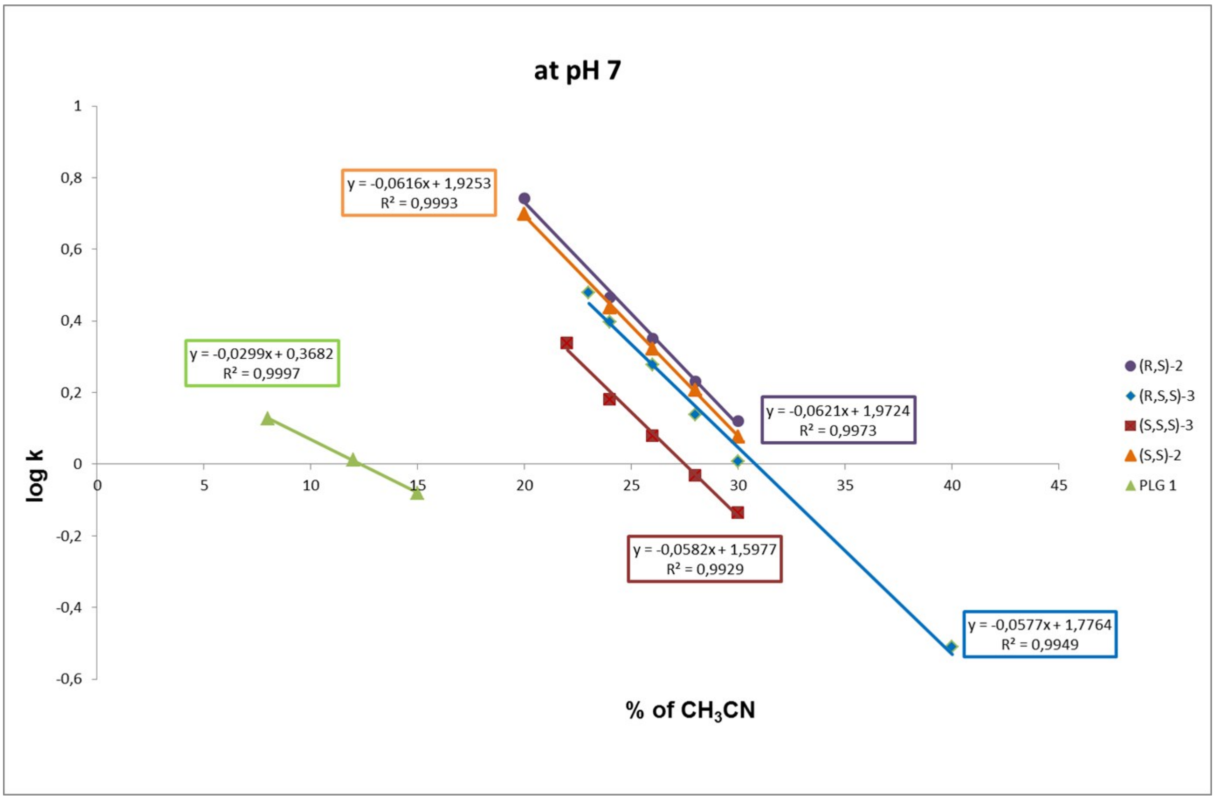

Supplement: RA-008-C8RA02511H-s001 [file RA-008-C8RA02511H-s001.pdf]
